# Cephalometric norms and esthetic profile preference for the Japanese: a systematic review

**DOI:** 10.1590/2177-6709.20.6.043-051.oar

**Published:** 2015

**Authors:** Caroline Nemetz Bronfman, Guilherme Janson, Arnaldo Pinzan, Thais Lima Rocha

**Affiliations:** 1 PhD resident in Orthodontics, Universidade de São Paulo (USP), School of Dentistry, Bauru, São Paulo, Brazil; 2 Full professor, Universidade de São Paulo (USP), School of Dentistry, Department of Orthodontics, Bauru, São Paulo, Brazil; 3 Associate professor, Universidade de São Paulo (USP), School of Dentistry, Department of Orthodontics, Bauru, São Paulo, Brazil; 4 PhD resident in Orthodontics, Universidade de São Paulo (USP), School of Dentistry, Bauru, São Paulo, Brazil

**Keywords:** Japan, Face, Dental radiography, Review

## Abstract

**Objective:**

To determine the cephalometric parameters and esthetic preferences of a pleasant face for the Japanese population.

**Methods:**

For the present study, the following databases were accessed: PubMed, Embase, Scopus and Web of Science. Initial inclusion criteria comprised studies written in English and quoting cephalometric norms and/or facial attractiveness in Japanese adults. No time period of publication was determined. The quality features evaluated were sample description, variables analyzed and how cephalometric standards or facial profile were evaluated.

**Results:**

Initially, 60 articles were retrieved. From the selected studies, 13 abstracts met the initial inclusion criteria. They were divided into two groups; seven articles were included in Group I and six articles in Group II, according to the criteria of evaluation: cephalometric or facial analyses.

**Conclusion:**

Japanese are characterized by having a less convex skeletal profile, bilabial protrusion, less prominent nose, more retruded chin and protruded mandibular incisor. Despite living in a society with homogeneous patterns, they seem to get an esthetic preference for white-like features. Therefore, in addition to ethnic normative values, patient's preferences to establish individual treatment plans should always be considered.

## Resumo

**Objetivo:**

determinar os padrões cefalométricos e a preferência estética do perfil considerado agradável para a população japonesa.

**Métodos:**

a literatura foi pesquisada a partir das seguintes bases de dados: PubMed, Embase, Scopus e Web of Science. Os critérios iniciais de inclusão foram artigos escritos em inglês, contendo assuntos como norma cefalométrica e/ou atratividade facial em japoneses adultos; nenhuma limitação quanto ao ano de publicação foi imposta. As características da amostra, as variáveis utilizadas e como foram avaliados os padrões cefalométricos e o perfil facial deveriam ser descritos nos estudos.

**Resultados:**

inicialmente, foram encontrados 60 artigos. Desses, 13 resumos apresentavam os critérios iniciais de inclusão e foram selecionados. Os trabalhos foram divididos em dois grupos, sendo incluídos 7 artigos no Grupo I e 6 artigos no Grupo II, de acordo com os critérios de avaliação: cefalometria ou análise facial.

**Conclusões:**

os japoneses caracterizam-se por apresentar um perfil esquelético menos convexo, lábios mais protruídos, um nariz menos proeminente, o mento mais retrusivo e uma maior projeção dos incisivos inferiores e, mesmo pertencendo a um grupo racial com padrões mais homogêneos, adquiriram um gosto estético por características mais internacionalizadas de leucodermas. Sendo assim, apesar de existirem valores normativos específicos para cada grupo racial, a preferência do paciente deverá sempre ser levada em consideração para se estabelecer um plano de tratamento individualizado.

## INTRODUCTION

Anatomists and physical anthropologists generally classify men into various racial groups based on their cephalometric features.^1^

Currently, metropolitan areas have a more diverse population, emphasizing the need to recognize that a single standard of facial esthetics may not be appropriate when making diagnostic and treatment planning decisions for patients with diverse racial and ethnic backgrounds.^2^

The Japanese population is a well-defined and homogeneous group with features that are proper even when compared with other Asian groups. Japanese subjects have more proclined incisors, thicker soft tissues, a more projected midface and a flat facial profile.^3^ Nowadays, an increasing number of Japanese are looking for orthognathic and orthodontic treatment and plastic surgery. Therefore, it has become important to determine the cephalometric parameters of hard and soft tissues for this ethnic group.^4^ Furthermore, orthodontists and surgeons should recognize these differences when interpreting measurements.^3^

The purpose of orthodontic treatment is to achieve a proper and functional occlusion combined with a well-balanced and esthetically pleasing facial appearance. Consensus is comparatively easy to achieve regarding occlusion. One way of expressing that consensus is known as "the six keys for normal occlusion", as proposed by Andrews.^5^ However, it is sometimes hard to define the treatment goal based on esthetic profile because no single facial type is believed to be attractive by all. Facial attractiveness might be related to several factors: ethnic group, age, sex, region and professional background. In particular, ethnic and racial differences play a major role in judging facial esthetics. Such judgments might be affected by differences in skeletal pattern among various ethnic groups. Thus, it is important to know the facial preferences of each ethnic group before orthodontic treatment.^6^

There are many studies about cephalometric norms and well-balanced faces in the Japanese population,^1^^,^ 2 ^,^ 4 ^,^ 6 ^-^ 16 but up to date, none of these studies have compared the inter-relationship between the bone pattern displayed by this ethnic group and its esthetic preferences for a pleasant face. People who are potential candidates for orthodontic treatment are likely to be profoundly influenced by the media, including the Internet, magazines, television and newspapers. Worldwide communication provides daily reinforcement for facial stereotypes and these are the major reasons why the perception of beauty might be changing to a more internationally pleasing one, thereby unifying preferences. This systematic review aims to determine the cephalometric parameters and esthetic preferences of a pleasant face for the Japanese population.

## MATERIAL AND METHODS

Using "cephalometric", "Japanese", "norms", and "profile" as keywords, research was conducted until February 2014 in the following electronic databases: PubMed, Embase, Scopus and Web of Science. To ensure that the research would encompass all studies related to the topic, the keywords were used, as follows: cephalometric AND Japanese AND (norms OR profile). Cochrane database was investigated for a systematic review on the subject and no data were found.

To identify potential articles, the initial research was performed by title. Initial inclusion criteria were studies written in English and quoting cephalometric norms and/or facial attractiveness in Japanese adults. No limitation on the year of publication of the studies was imposed. This selection process was independently conducted by two researchers. Thereafter, the articles from the selected titles were evaluated by abstract and independently valued by the examiners. Interexaminer conflicts were solved by discussion on each article, so as to reach a consensus regarding which articles fulfilled the main selection criteria.

The ultimately selected articles were then classified based on the following quality features: sample description, description of the analyzed variables and description of how the cephalometric standards or facial profiles were evaluated.

Sample description was considered adequate when the author clearly established the evaluated sample. The inclusion criteria were: adult Japanese, with an ANB angle between 2° and 5°, good facial symmetry, normal occlusion with minor or no crowding, all teeth present except third molars, no previous orthodontic treatment and no prosthetic replacement of teeth.

The analyzed variables were adequate when the article showed which angular and linear variables were evaluated and from which cephalometric analysis they were from.

The study was considered appropriate when the author described with which ethnic group the Japanese were compared to, and when their profiles were evaluated, in addition to examiners description.

Afterwards, the articles were divided into two groups: Group I (studies on cephalometric norms) and Group II (studies on facial profile). Then, qualification features were created to classify the articles based on the scientific weight.^17^ Articles with most of the qualification features, earning 5 to 6 points, were classified as with high quality; articles with some of them, earning 3 to 4 points, as average; and those with few characteristics, earning 2 points or less, were classified as with low quality.

## RESULTS

**Figure 1:**
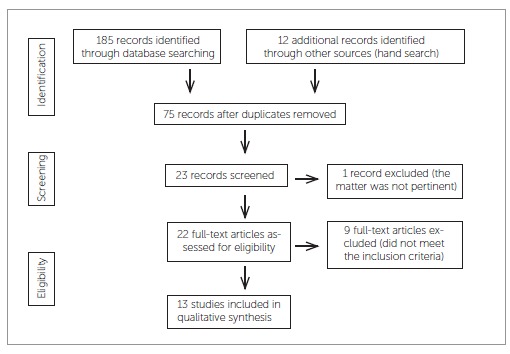
- Flow diagram of information through the different phases of article selection.

After database search, 60 articles were found from PubMed, 37 from Embase, 36 from Scopus and 52 from Web of Science, but some of them were repeated. From hand search, 12 studies were identified. The entire search strategy, excluding the repeated articles, resulted in 22 abstracts (Fig 1 andTable 1). Studies retrieved from 1965 up to the present demonstrate that the interest in different racial groups still attracts a number of orthodontists.

**Table 1:** - Search terms and number of articles processed in each selection phase.

**Database**	**Keywords**	**Results**	**Selected**	**% of total selected abstracts**
PubMed	Cephalometric, Japanese, norms or profile	60	7	5
Embase	Cephalometric, Japanese, norms or profile	37	8	5
Scopus	Cephalometric, Japanese, norms or profile	36	11	6
Web of Science	Cephalometric, Japanese, norms or profile	52	11	9
Cochrane	Cephalometric, norms, Japanese	0	0	0
Hand search		12	6	6
Total		23*	22*	13*

* The final sum corresponds to the total references without repetition.

Thirteen articles met the initial inclusion criteria: cephalometric norms and facial profile. They were divided into two groups. The division in groups and their respective qualification features are shown in Tables 2 and 3. After quality feature analysis, all articles were classified as high-quality level in Group I; and four articles were classified as high-level, and two as average-quality level in Group II.

**Table 2:** - Group I: quality features analyzed from studies on cephalometric norms.

**Article**	**Year of publication**	**Quality feature**					
		**Ethnic group compared**	**Sample size**	**Balanced sample of man and woman**	**Provides table with measurements**	**Measures soft and hard tissues**	**Inclusion criteria of sample**
Reitz et al^13^	1973	X	X	X	X	X	X
Miyajima et al^2^	1996	X	X	X		X	X
Alcade et al^4^	1998	X	X	X	X	X	X
Alcade et al^7^	2000	X	X	X	X		X
Scavone et al^15^	2006	X	X	X	X		X
Ioi et al^9^	2007	X	X	X	X	X	X
Shindoi et al^3^	2013	X	X		X	X	X

**Table 3:** - Group II: quality features analyzed from studies on facial profile.

**Article**	**Year of publication**	**Quality feature**					
		**Kind of raters**	**Sample size**	**Balanced sample of man and woman**	**Elimination of distracting variables**	**Measures soft and hard tissues**	**Inclusion criteria of sample**
Mantzikos T.^11^	1998	X	X	X	X		
Ioi et al^8^	2005	X	X	X	X	X	X
Ioi et al^10^	2008	X	X	X	X	X	X
Nomura et al^12^	2009		X	X	X	X	X
Kuroda et al^6^	2009	X	X			X	X
Shimomura et al^16^	2011	X	X	X	X	X	X

### Comparison of cephalometric norms

In Group I, cephalometric parameters of adult Japanese with normal occlusion and well-balanced faces were evaluated in seven studies. In these studies, cephalometric values were obtained from lateral cephalometric radiographs of different ethnic groups and compared with each other. Only one article used facial-profile photographs to set landmarks and measure soft tissue profile variables.^15^

Each article used a specific cephalometric analysis to compare the Japanese with another racial group. Angular and linear measurements used in these studies derived from different cephalometric analyses.

In all selected articles, cephalometric radiograph tracings were made by hand, traced and digitized by a single author in order to eliminate interexaminer variability.

Table 4 shows the cephalometric analyses used and the ethnic groups compared to the Japanese.

**Table 4:** - Characterization of cephalometric analysis and ethnic group compared.

**Article**	**Year of publication**	**Cephalometric landmarks**	**Ethnic group compared with the Japanese**
Reitz et al^13^	1973	The authors describe lines and planes to create 17 angular measurements.	Caucasian - American
Miyajima et al^2^	1996	McNamara	European - American
Alcade et al^4^	1998	Burstone and Leagan	White American
Alcade et al^7^	2000	Ricketts Epker et al Legan and Burstone Holdaway	White
Scavone et al^15^	2006	Arnett et al	White American
Ioi et al^9^	2007	Riolo et al McNamara Miyajima et al Legan and Burstone Bishara Burstone and Marcotte	Caucasian
Shindoi et al^3^	2013	Arnett et al	White

Data extracted from the articles were separated according to individuals' sex and grouped according to skeletal or dental relationships as well as soft tissue analysis, as shown in Tables 5 and 6.

**Table 5:** - Comparison of cephalometric norms between Japanese and Caucasian men.

**Japanese men**	**Reitz et al,^13^ 1973**	**Miyajima et al,^2^ 1996**	**Alcalde et al,^4^ 1998**	**Alcalde et al,^7^ 2000**	**Scavone et al,^15^ 2006**	**Ioi et al,^9^ 2007**	**Shindoi et al,^3^ 2013**	
Skeletal relationship	More retruded mandibular A-P position						X	
	Shorter maxilla in A-P dimension			X	X			X
	Reduced midfaces		X					
	Smaller facial axis angle		X					
	Larger Frankfort to mandibular plane angle						X	
Soft tissue	Bilabial protrusion	X	X	X	X	X	X	X
	Smaller nasolabial angle		X				X	
	Less prominent nose	X			X	X		
	Retruded chin				X			X
	Larger labiomental sulcus						X	
	Smaller Z-angle						X	
Dental relationship	More protruded lower incisor		X				X	X

**Table 6:** - Comparison of cephalometric norms between Japanese and Caucasian women.

**Japanese women**		**Reitz et al,^13^ 1973**	**Miyajima et al,^2^ 1996**	**Alcalde et al,^4^ 1998**	**Alcalde et al,^7^ 2000**	**Scavone et al,^15^ 2006**	**Ioi et al,^9^ 2007**	**Shindoi et al,^3^ 2013**
Skeletal relationship	More retruded mandibular A-P position						X	
	Shorter maxilla in A-P dimension			X	X			X
	Reduced midfaces		X					
	Larger lower facial height						X	X
	Steeper mandibular plane angle		X					
Soft tissue	Bilabial protrusion	X	X	X	X	X	X	X
	Smaller nasolabial angle		X					
	Less prominent nose	X			X	X		
	Retruded chin				X	X		
Dental relationship	More protruded lower incisor		X				X	X

The differences found in Japanese when compared with white standards are:

» In anteroposterior dimension: the Japanese showed a more retruded mandibular position, retrognathic maxilla, more protruded mandibular incisors and lip position, and reduced nasal projection.

» In vertical dimension: the Japanese showed reduced midfaces and larger lower facial height.

### Comparison of facial profile

In Group II, six articles evaluated the components of a well-balanced Japanese facial profile. The studies assessed the most favored or most well-balanced profile selected by different methods. Japanese silhouettes as well as profile photographs were based on Japanese adults with a harmonious facial profile, and the images were modified creating profiles with more or less protruded lips, or by horizontally altering middle and lower facial thirds. To avoid subjective considerations, four articles used facial silhouettes,^8^^,^ 10 ^,^ 12 ^,^ 16 whereas the other two^6^^,^ 11 used facial profile photographs in which distracting variables, such as hairstyle and make-up, were eliminated.

Table 7 shows the methods used to evaluate profiles, the types of examiners and the results.

**Table 7:** - Methods, examiners and results of evaluation of Japanese profiles.

**Article**	**Year of publication**	**Methods to evaluate the profiles**	**Examiners**	**Results**
Mantzikos et al^11^	1998	Five facial profile types were computer-generated to represent distinct facial types.	Japanese cultural and educational background that have immigrated from Japan within the past 5 years.	The profiles preferred were (in descending order): orthognatic, bimaxillary dentoalveolar retrusion, bimaxillary dentoalveolar protrusion, mandibular retrognathism and mandibular prognathism.
Ioi et al^8^	2005	Series of facial silhouettes with varying anteroposterior lip position.	Japanese orthodontists and young adult Japanese dental students.	Both orthodontists and students preferred a profile with slightly retruded lips.
Ioi et al^10^	2008	Series of facial silhouettes with varying anteroposterior lip position.	Young Korean and Japanese adults.	Both the Korean and Japanese tended to prefer slightly more retruded lip position.
Nomura et al^12^	2009	Silhouette profiles with various distances from lip to E-line.	Lay judges of European American, Hispanic American, Japanese and African.	All judges preferred lips located posterior to the E-line.
Kuroda et al^6^	2009	Profile images with point B and Menton anteriorly or distally moved by software.	Male and female Japanese laypeople.	Moderate mandibular retrusion was the most favored profile. A slight mandibular retrusion is more favorable than the mean image, and mandibular protrusion is less attractive.
Shimomura et al^16^	2011	Series of facial silhouettes with varying anteroposterior lip position.	Male and female orthodontic Japanese patients.	Patients tended to prefer a lip position that was slightly retruded compared with the average facial profile for both men and women.

According to the results, the Japanese preferred a retruded profile with moderate mandibular and lip retrusion.

## DISCUSSION

### Group I

Cephalometric norms for the Japanese have been studied and extensively used for research and clinical purposes. In order to determine the differences in skeletal relationship, dental relationship and soft tissue analysis, seven articles were used in this systematic review. All selected studies compared a group of non growing Japanese (males and females) to white samples.

The Japanese showed a less convex skeletal profile due to the retruded position of the maxilla and mandible. They presented a significantly less prominent nose^7^ and the upper and lower lips anteriorly positioned in all studies, which agreed with the concept of bilabial protrusion.^2^^,^ 3 ^,^ 4 ^,^ 7 ^,^ 9 ^,^ 13 ^,^ 15

Two articles^7^^,^ 13 analyzed and compared soft tissue measurements while one compared hard tissue measurements.^13^ Males and females adults were included in the samples, but data were not segregated according to sex. These articles showed that Japanese subjects have a less convex skeletal profile, less proeminent nose, anteriorly positioned upper and lower lips and a retruded chin, thereby increasing the H-angle. The H-angle is the angle between the H-line (soft tissue pogonion - upper lip) and soft tissue facial line (soft tissue nasion to soft tissue pogonion).

Five articles^2^^,^ 3 ^,^ 4 ^,^ 9 ^,^ 15 grouped data according to sex and are discussed as follows.

### Japanese males

Skeletally, Japanese males showed a vertically larger middle third as well as larger posterior dental height.^4^ The maxilla was shorter in the anteroposterior dimension,^2^^,^ 7 with a more retruded chin and mandible.^9^ They also had a steeper Frankfort-to-mandibular-plane angle.^9^

Regarding soft tissues, Japanese males exhibited bilabial protrusion,^2^^,^ 4 ^,^ 9 ^,^ 15 smaller noses,^15^ less proeminent chin,^4^ as well as posteriorly positioned maxilla and mandible in relation to the glabella, leading to less convex facial form.^4^ They also presented a smaller nasolabial angle,^2^^,^ 3 ^,^ 9 ^,^ 15 larger labiomental sulcus,^9^ smaller Z-angle^9^ and a thinner base of the upper lip.^9^

Regarding dental relationships, there was greater protrusion of mandibular incisors.^2^^,^ 9

### Japanese females

Skeletally, Japanese females showed anteroposteriorly shorter maxilla, greater anterior middle third of the face^4^ and significantly larger lower facial height.^9^ The midface and the facial axis angle were smaller,^2^^,^ 9 and the Frankfort to mandibular plane angle was larger,^9^ with a more retruded mandible and chin.^9^

Regarding soft tissues, Japanese females exhibited bilabial protrusion^2^^,^ 4 ^,^ 9 ^,^ 15 and a less proeminent chin.^4^ The nasolabial angle was more acute,^2^ with a smaller nasal projection.^15^ There was no difference in the Z-angle between Japanese and Caucasian females,^9^and racial differences in the cant of the upper lip were less obvious in women than in men.^2^

Regarding dental relationships, there was greater protrusion of mandibular incisors.^2^^,^ 3 ^,^ 9 The distance of mandibular incisors and molars to the mandibular plane was significantly larger than in Caucasian subjects. These differences might be attributed to longer lower face height in Japanese females.^9^

### Sexual dimorphism

Sexual dimorphism was found in Japanese adults, with Japanese males showing longer anteroposterior cranial base length and longer vertical skeletal and dental values than the female group. Longer maxillary and mandibular measurements and larger gonial angle were found in Japanese men. Japanese women had a more obtuse angle between occlusal and mandibular planes,^4^ and had a more projected midface and convex profile .^3^ Despite sexual differences in some dentoskeletal variables, there were no sexual differences regarding soft-tissue variables.^15^

### Group II

The average anteroposterior lip position in Japanese adults is regarded to be more protrusive than that of white people. Because one of the goals of orthodontic treatment is to create an esthetic profile, it is important to study the Japanese esthetic preferences because different racial groups have different perceptions of attractiveness.

In Group II, the studies were conducted with various types of examiners, such as: Japanese laypeople,^6^^,^ 11 Japanese orthodontic patients,^16^ lay judges from different ethnicities,^10^^,^ 12 Japanese orthodontists and dental students,^8^ representing a wide variety of esthetic preferences of a particular population. These data suggest that the Japanese prefer a retruded or a straight profile, even though Japanese profiles have been characterized as being more protrusive due to typically protruded incisors. Thus, orthodontic treatment should consider patient's opinion to establish individual treatment plans.

Some studies were performed to determine how sex, age or different ethnicity influences the perception of beauty.

### Sex

Orthodontists and dental students examiners preferred a slightly more retruded profile for both men and women; but for Japanese females, even a more retruded lip position is preferable.^8^

### Age

There was no age difference regarding the preference for male profile. However, examiners over 30 years old preferred a more retruded lip position than those aged between 15 to 19 and 20 to 29 years old for the female profile.^16^

### Ethnicity

Examiners' race had significant influence on preference judgement of lip profile. American, Japanese and African preferred lip position posterior to the E-line, but American and Japanese examiners preferred a more retruded lip profile than did the African.^12^

Korean and Japanese people have similar cultural backgrounds and both tended to prefer slightly more retruded lip positions.^10^

### According to the profile

According to Mantzikos,^11^ an orthognathic profile was most preferred and mandibular protrusion was the least favored off all profiles in the Japanese population. Mandibular retrusion was generally more favored than mandibular protrusion, but the Japanese's favorite profile depends much more on lip position than on chin position.^6^

## CONCLUSION

» Japanese adults are characterized by having a less convex skeletal profile, bilabial protrusion, less proeminent nose, more retruded chin and protruded mandibular incisors, when compared to a white population.
» Although anteroposterior lip position in Japanese adults is more protrusive, they prefer a more retruded profile.
» Orthodontists should always consider, in addition to ethnic normative values, patient's preferences before establishing individual treatment plans.
